# Is mesh pore size in polypropylene meshes associated with the outcome in Lichtenstein inguinal hernia repair: a registry-based analysis of 22,141 patients

**DOI:** 10.1007/s10029-024-03029-5

**Published:** 2024-05-01

**Authors:** H. C. Albrecht, M. Trawa, F. Köckerling, D. Adolf, M. Hukauf, H. Riediger, S. Gretschel

**Affiliations:** 1 Department of General, Visceral, Thoracic and Vascular Surgery, Faculty of Health Science Brandenburg, Brandenburg Medical School, University Hospital Ruppin-Brandenburg, Fehrbelliner Str. 38, 16816 Neuruppin, Germany; 2 Department of Surgery, Hernia Center, Academic Teaching Hospital of Charité Medical School, Vivantes Humboldt-Hospital Berlin, Berlin, Germany; 3 StatConsult GmbH, Magdeburg, Germany

**Keywords:** Mesh pore size, Lichtenstein hernioplasty, Inguinal hernia, Outcome hernia repair, Chronic pain, Hernia recurrence

## Abstract

**Introduction:**

Experimental data show that large-pored meshes reduce foreign body reaction, inflammation and scar bridging and thus improve mesh integration. However, clinical data on the effect of mesh porosity on the outcome of hernioplasty are limited. This study investigated the relation of pore size in polypropylene meshes to the outcome of Lichtenstein inguinal hernioplasty using data from the Herniamed registry.

**Methods:**

This analysis of data from the Herniamed registry evaluated perioperative and 1-year follow-up outcomes in patients undergoing elective, primary, unilateral Lichtenstein inguinal hernia repair using polypropylene meshes. Patients operated with a non-polypropylene mesh or a polypropylene mesh with absorbable components were excluded. Polypropylene meshes with a pore size of 1.0 × 1.0 mm or less were defined as small-pored meshes, while a pore size of more than 1.0 × 1.0 mm was considered large-pored.

Unadjusted analyses and multivariable analyses were performed to investigate the relation of pore size of polypropylene meshes, patient and surgical characteristics to the outcome parameters.

**Results:**

Data from 22,141 patients were analyzed, of which 6853 (31%) were operated on with a small-pore polypropylene mesh and 15,288 (69%) with a large-pore polypropylene mesh. No association of mesh pore size with intraoperative, general or postoperative complications, recurrence rate or pain requiring treatment was found at 1-year follow-up. A lower risk of complication-related reoperation tended to be associated with small-pore size (*p* = 0.086). Furthermore, small-pore mesh repair was associated with a lower risk of pain at rest and pain on exertion at 1-year follow-up.

**Conclusion:**

The present study could not demonstrate an advantage of large-pore polypropylene meshes for the outcome of Lichtenstein inguinal hernioplasty.

## Introduction

Inguinal hernioplasty is one of the most frequently performed surgical procedures in which synthetic mesh implants are nowadays used for standard repair, as recurrence rates are significantly lower compared to non-mesh repair [1]. However, several complications have been associated with mesh repairs, most commonly chronic postoperative inguinal pain (CPIP), which can occur in up to 50% of patients [2, 3]. Due to its frequency, this complication has a significant impact on patients’ quality of life [2]. Furthermore, mesh implants have been assumed to affect the spermatic cord structures and, in rare cases, cause dysejaculation, sexual pain, and orchialgia [4–6].

However, potential complications after mesh repair are influenced by several factors, among which surgical technique and mesh material parameters are considered relevant [7, 8].

Synthetic mesh implants differ in terms of raw material, mechanical and structural parameters [5]. In the latter, mesh weight and porosity are in focus with regard to biocompatibility and outcomes after inguinal hernioplasty.

The postulated advantage of light weight meshes (<50 g/m^2^) with regard to CPIP [3, 9–12] due to lower foreign body reactions, inflammation and fibrosis could not be consistently confirmed in all studies [13–15]. However, since the mesh weight depends significantly on specific density of the polymer, it is not considered the only relevant predictor for tissue reaction [7, 8, 13]. Several studies have shown that porosity is instead an even more important factor for the biocompatibility of synthetic meshes. Large pores (>1 mm) improve tissue ingrowth by reducing inflammatory infiltration, connective tissue and scar bridging [8, 16, 17]. The extent to what these findings transfer to the results of inguinal hernioplasty, both in studies and in everyday clinical practice, is still controversial [2, 9, 14].

The aim of this study was to investigate the association of pore size in polypropylene meshes with the outcome of Lichtenstein inguinal hernioplasty using data from the Herniamed registry.

## Patients and methods

Herniamed is an internet-based hernia registry in which hospitals and independent surgeons in Germany, Austria and Switzerland can voluntarily enter data on their routine hernia operations [18, 19]. A contract is concluded with each participating hospital and each participating surgeon, in which the latter two parties undertake to enter all data on hernia repairs completely and correctly in the Herniamed register. For routine procedures, surgeons choose one of the meshes available on the market.

However, in order for a patient to be included in the Herniamed registry, the patient must sign a special declaration of consent in which the patient agrees to the data documentation and follow-up by the treating hospital or surgeon. If this special declaration of consent is not available, the patient may not be included in the Herniamed registry. As part of the information patients receive about participating in the Herniamed registry, they are also encouraged to inform the treating hospital or surgeon of any problems or complications that occur after hernia surgery.

If problems or complications arise after the operation, the patient can contact the treating hospital or surgeon at any time and request a clinical examination [18, 19]. All intra- and postoperative complications as well as complication-related reoperations are recorded up to 30 days after the operation. After 1, 5 and 10 years, all patients and their general practitioner receive a questionnaire from the hospital or the treating surgeon in which they are asked about pain at rest, pain on exertion, chronic pain requiring treatment, protrusions in the groin area or recurrence. Patients are also asked again whether any postoperative complications have occurred. If the patient or the general practitioner reports a relevant finding, the patient may be requested to attend for further diagnostic examination [18, 19]. Haapaniemi et al. [20] showed that participation in the registry and follow-up by questionnaire and selective physical examination provide a solid basis for quality control.

Data from the Herniamed database collected prospectively between January 5, 2009 and December 1, 2021 were used for the analysis. Inclusion criteria were an age of 16 years or older and an elective, primary, unilateral Lichtenstein inguinal hernia repair performed with one of the selected polypropylene meshes (Table 1) by November 30, 2021. Non-polypropylene meshes and polypropylene meshes with absorbable components were excluded.

**Table 1:** Polypropylene meshes used for Lichtenstein repair

Mesh					
Small pore (≤ 1.0 × 1.0 mm)	*n*	%	Large pore (> 1.0 × 1.0 mm)	*n*	%
Optilene LP 36 g/m^2^; 1.0 × 1.0 mm	4140	60.41	DynaMesh-LICHTENSTEIN 68 g/m^2^; 2.6 × 2.6 mm	3651	23.88
Prolene 76 g/m^2^; 1.0 × 1.0 mm	1162	16.96	Optilene mesh 60 g/m^2^ 1.5 × 1.5 mm	2518	16.47
TiMesh Light 35 g/m^2^; 1.0 × 1.0 mm	1050	15.32	Dynamesh Endolap 78 g/m^2^; 2.6 × 2.6 mm	1922	12.57
Premilene 82 g/m^2^; 0.8 × 0.8 mm	325	4.74	Bard Soft mesh 42 g/m^2^; 6.29 × 6.29 mm	1918	12.55
TiMesh strong 65 g/m^2^; 1.0 × 1.0 mm	90	1.31	Parietene Standard 88 g/m^2^; 2.0 × 2.6 mm	1677	10.97
TiMesh extra light 16 g/m^2^; 1.0 × 1.0 mm	57	0.83	Dynamesh PP standard 70 g/m^2^; 1.6 × 1.6 mm	831	5.44
3D Max Mesh 104.5 g/m^2^; 0.55 × 0.55 mm	29	0.42	Optilene Mesh elastic 48 g/m^2^; 3.6 × 2.8 mm	813	5.32
			Dynamesh PP light 37 g/m^2^; 2.4 × 2.4 mm	759	4.96
			Parietene makroporös 46/m^2^; 2.8 × 2.5 mm	625	4.09
			TiO2Mesh 47 g/m^2^; 2.8 × 2.8 mm	292	1.91
			Tilene blue 24 g/m^2^; 3.0 × 3.0 mm	243	1.59
			3D Max Light Mesh 42 g/m^2^; 6.29 × 6.29 mm	36	0.24
			Parietene light 36/m^2^; 1.6 × 1.5 mm	3	0.02
Total	6853	100.00		15,288	100.00

The polypropylene meshes with a pore size of 1.0 × 1.0 mm or less were defined as small-pore meshes. Accordingly, meshes with a pore size of more than 1.0 × 1.0 mm were considered large pored.

Exclusion criteria were incomplete documentation (e.g., missing age, weight, and duration of surgery), follow-up examinations outside the specified time period and data from the deactivated centers which could not be followed up before deactivation. In addition, femoral and scrotal hernias and operations with combined mesh fixation methods (e.g., staple and adhesive) were excluded. In addition, patients operated with non-polypropylene meshes or polypropylene meshes with absorbable components were excluded.

After plausibility and inclusion criteria were checked, the data were first examined univariately for differences between the pore size groups with regard to all patient and surgical characteristics and outcome parameters. Finally, binary logistic regression models were created for all outcome parameters with all potential influencing factors. The binary variables general, intra- or postoperative complications or a risk factor were deemed present each as soon as at least one corresponding single item was present.

In the univariate analysis, the small- and large-pore groups were compared with each other. All categorical patient data were presented as absolute and relative frequencies for these categories in contingency tables and tested via Chi-square test. For continuous parameters, mean and standard deviation were presented and the robust *t*-test (Satterthwaite) was used for normally distributed data.

The association of patient and surgical characteristics with the outcome parameters (general, intra- and postoperative complications, complication-related reoperations, recurrences as well as pain at rest, pain on exertion and pain requiring treatment after 1 year) was investigated using the binary logistic regression model.

All analyses were performed using SAS 9.4 software (SAS Institute Inc., Cary, NC, USA) and are deliberately considered at the full significance level of 5%, i.e., no correction is made for multiple testing.

## Results

**Fig. 1:**
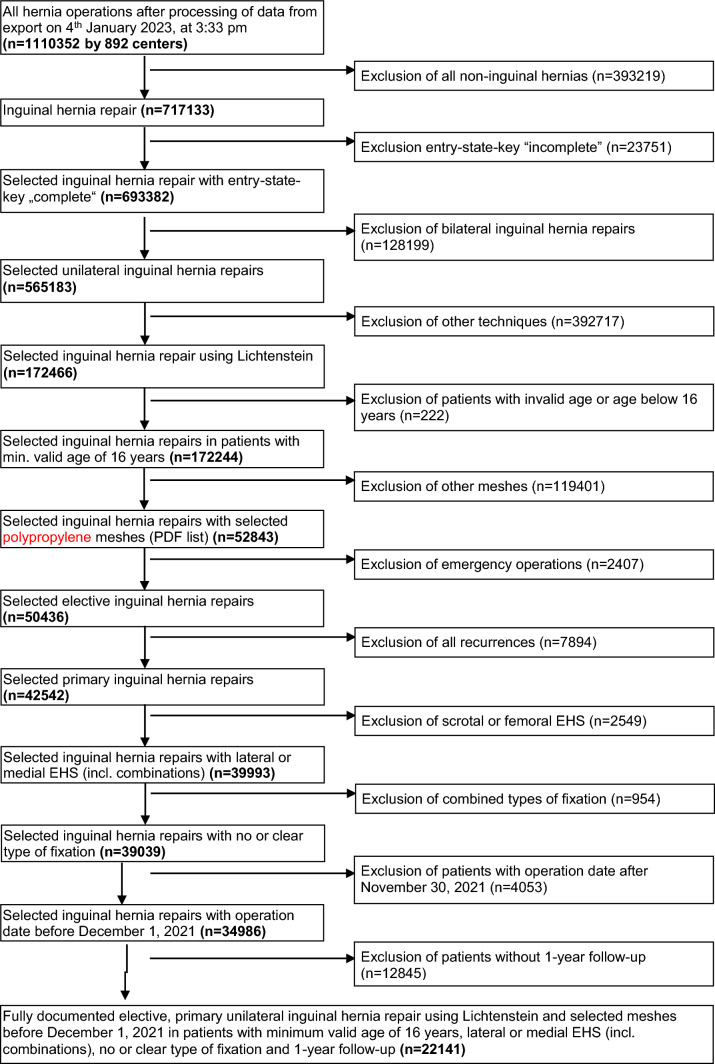
Flowchart of patient inclusion

Between January 2009 and January 2023, 1,110,352 patient records were prospectively enrolled in the Herniamed registry. The data of 22,141 patients were included in the present study with regard to inclusion criteria, plausibility and completeness of follow-up (Fig. 1).

Of the latter, 6853 patients (31%) were operated on with a small-pore mesh and 15,288 patients (69%) with a large-pore mesh in Lichtenstein repair.

### Non-adjusted analyses

With regard to patient and surgical characteristics, there were significant differences between the small- and large-pore polypropylene meshes for most parameters (Table 2). For example, the patients in whom a large-pored mesh was used were older and had a higher proportion of heavyweight meshes. In addition, these patients had a higher proportion of ASA III/IV, risk factors, preoperative pain, larger defects and use of sutures.

**Table 2:** Non-adjusted analysis of patient and surgical characteristics

		Pore size				*p*
		Small		Large		
		*n*	%	*n*	%	
Age [years] N/[mean ± SD]		6853/67.7 ± 13.7		15,288/68.6 ± 13.4		< 0.001
BMI [kg/m^2^] N/[mean ± SD]		6830/25.7 ± 3.5		15,237/25.9 ± 3.7		< 0.001
Mesh weigh	≤ 50 g/m^2^	5247	76.6	4689	30.7	< 0.00
	> 50 g/m^2^	1606	23.4	10,599	69.3	
Sex	Male	6071	88.6	13,943	91.2	< 0.001
	Female	782	11.4	1345	8.8	
ASA	I	1061	15.5	2088	13.7	< 0.001
	II	3896	56.9	8029	52.5	
	III/IV	1896	27.7	5171	33.8	
Preoperative pain	No	2126	31.0	4507	29.5	< 0.001
	Yes	3822	55.8	9572	62.6	
	Unknown	905	13.2	1209	7.9	
Defect size (inguinal)	I (< 1.5 cm)	826	12.1	1457	9.5	< 0.001
	II (1.5–3 cm)	4129	60.3	8200	53.6	
	III (> 3 cm)	1898	27.7	5631	36.8	
EHS classification (inguinal)	Medial	1982	28.9	4453	29.1	0.473
	Lateral	3318	48.4	7277	47.6	
	Combined	1553	22.7	3558	23.3	
Fixation	No mesh fixation	107	1.6	234	1.5	< 0.001
	Tacks	11	0.2	33	0.2	
	Suture	6468	94.4	14,929	97.7	
	Glue	267	3.9	92	0.6	
Risk factors—total	Yes	2642	38.6	6325	41.4	< 0.001
	No	4211	61.4	8963	58.6	
COPD	Yes	633	9.2	1195	7.8	< 0.001
	No	6220	90.8	14,093	92.2	
Diabetes	Yes	639	9.3	1400	9.2	0.691
	No	6214	90.7	13,888	90.8	
Aortic aneurysm	Yes	54	0.8	169	1.1	0.029
	No	6799	99.2	15,119	98.9	
Immunosuppression	Yes	74	1.1	172	1.1	0.767
	No	6779	98.9	15,116	98.9	
Corticoids	Yes	81	1.2	223	1.5	0.102
	No	6772	98.8	15,065	98.5	
Smoking	Yes	611	8.9	1322	8.6	0.513
	No	6242	91.1	13,966	91.4	
Coagulopathy	Yes	173	2.5	515	3.4	<0 .001
	No	6680	97.5	14,773	96.6	
Antithrombotic medication	Yes	978	14.3	2669	17.5	< 0.001
	No	5875	85.7	12,619	82.5	
Anticoagulant medication	Yes	358	5.2	894	5.8	0.063
	No	6495	94.8	14,394	94.2	

The relation of the pore sizes of the meshes to the intraoperative, general and postoperative complications, complication-related reoperations, recurrences as well as pain at rest, exertion and pain requiring treatment in the 1-year follow-up is shown in Table 3.

**Table 3:** Non-adjusted analysis of perioperative complications and 1-year follow-up data

		Pore size				*p*
		Small		Large		
		*n*	%	*n*	%	
Intraoperative complications—total	Yes	48	0.7	106	0.7	0.953
	No	6805	99.3	15,182	99.3	
General complications—total	Yes	110	1.6	210	1.4	0.182
	No	6743	98.4	15,078	98.6	
Postoperative complications—total	Yes	271	4.0	578	3.8	0.534
	No	6582	96.0	14,710	96.2	
Complication-related reoperations	Yes	77	1.1	225	1.5	0.039
	No	6776	98.9	15,063	98.5	
Recurrence on 1-year follow-up	Yes	65	0.9	139	0.9	0.777
	No	6788	99.1	15,149	99.1	
Pain on exertion on 1-year follow-up	Yes	651	9.5	1614	10.6	0.016
	No	6202	90.5	13,674	89.4	
Pain at rest on 1-year follow-up	Yes	345	5.0	874	5.7	0.040
	No	6508	95.0	14,414	94.3	
Pain requiring treatment on 1-year follow-up	Yes	188	2.7	472	3.1	0.164
	No	6665	97.3	14,816	96.9	

In the unadjusted analysis, there was a significant disadvantage for patients in whom large-pored meshes were used in terms of complication-related reoperations as well as rest pain and pain on exertion in the 1-year follow-up.

### Multivariable analyses

The risk of intraoperative complications was associated with defect size (*p*=0.003), mesh fixation (*p* = 0.003) and ASA classification (*p* = 0.058) (Table 4). The results showed a lower complication rate for suture vs. glue fixation.

**Table 4:** Multivariable analysis for intraoperative complications

Variable	*p*-value	Categories	Odds ratio	LCL	UCL	*p*-value (pair-wise)
Defect size	0.003	III (> 3 cm) vs II (1.5–3 cm)	1.840	1.294	2.616	< 0.001
		II (1.5–3 cm) vs I (< 1.5 cm)	0.727	0.420	1.259	0.255
		III (> 3 cm) vs I (< 1.5 cm)	1.338	0.760	2.355	0.313
Fixation	0.003	Suture vs glue	0.259	0.127	0.530	< 0.001
		No mesh fixation vs glue	0.355	0.094	1.345	0.127
		No mesh fixation vs suture	1.369	0.433	4.328	0.593
		Tacks vs glue*	–	–	–	0.977
		No mesh fixation vs tacks*	–	–	–	0.979
		Suture vs tacks*	–	–	–	0.979
ASA	0.058	III/IV vs II	1.515	1.038	2.209	0.031
		II vs I	0.702	0.424	1.163	0.169
		III/IV vs I	1.064	0.598	1.891	0.833
BMI [5-point OR]	0.139		1.168	0.951	1.435	
Preoperative pain	0.391	Unknown vs no	0.645	0.326	1.275	0.207
		Yes vs no	0.854	0.606	1.203	0.366
		Yes vs unknown	1.324	0.685	2.560	0.403
Sex	0.625	Female vs male	0.861	0.473	1.568	
EHS classification	0.650	Medial vs lateral	0.832	0.564	1.229	0.356
		Medial vs combined	0.877	0.563	1.367	0.562
		Lateral vs combined	1.054	0.705	1.575	0.799
Age [10-year OR]	0.680		1.030	0.895	1.186	
Risk factors	0.843	Yes vs no	0.965	0.680	1.371	
Mesh weight	0.887	≤ 50 g/m^2^ vs > 50 g/m^2^	1.026	0.719	1.466	
Pore size	0.993	Small vs large	0.998	0.677	1.473	

*LCL* lower confidence limit, *UCL* upper confidence limit

* No valid information available as categories are not sufficiently populated

The risk of general complications was significantly increased with high ASA classification (*p*<0.001), older age (*p* = 0.004) and the presence of risk factors (*p* = 0.038) (Table 5).

**Table 5:** Multivariable analysis for general complications

Variable	*p*-value	Categories	Odds ratio	LCL	UCL	*p*-value (pair-wise)
ASA	< 0.001	III/IV vs II	1.943	1.501	2.515	< 0.001
		III/IV vs I	1.542	0.983	2.420	0.060
		II vs I	0.794	0.522	1.207	0.279
Age [10-year OR]	0.004		1.172	1.052	1.307	
Risk factors	0.038	Yes vs no	1.292	1.015	1.645	
Mesh weight	0.138	≤ 50 g/m^2^ vs > 50 g/m^2^	1.204	0.942	1.539	
Defect size	0.154	III (> 3 cm) vs II (1.5–3 cm)	1.247	0.980	1.586	0.073
		III (> 3 cm) vs I (< 1.5 cm)	1.347	0.855	2.122	0.199
		II (1.5–3 cm) vs I (< 1.5 cm)	1.080	0.698	1.673	0.729
Pore size	0.241	Small vs large	1.169	0.900	1.518	
EHS classification	0.666	Lateral vs combined	0.885	0.671	1.168	0.387
		Medial vs combined	0.898	0.664	1.214	0.484
		Medial vs lateral	1.015	0.774	1.330	0.915
BMI [5-point OR]	0.741		1.027	0.878	1.200	
Preoperative pain	0.821	YES vs no	1.085	0.840	1.400	0.532
		Unknown vs no	1.045	0.682	1.601	0.841
		Yes vs unknown	1.038	0.699	1.543	0.852
Fixation	0.973	No mesh fixation vs tacks	0.613	0.067	5.643	0.666
		No mesh fixation vs suture	0.828	0.306	2.238	0.710
		No mesh fixation vs glue	0.813	0.225	2.936	0.752
		Suture vs tacks	0.741	0.101	5.422	0.768
		Tacks vs glue	1.325	0.154	11.373	0.797
		Suture vs glue	0.982	0.428	2.250	0.965
Sex	0.989	Female vs male	1.003	0.682	1.475	

*LCL* lower confidence limit, *UCL* upper confidence limit

A similar relation was found for postoperative complications. Furthermore, preoperative pain compared to unknown pain status (*p* = 0.003), large hernia defect (*p* = 0.013), lightweight mesh (*p* = 0.013), female gender (*p* = 0.010) and high BMI (*p* = 0.027) were identified as being related to a higher risk for postoperative complications (Table 6). However, a medial EHS classification was associated with a lower complication rate compared to a lateral classification.

**Table 6:** Multivariable analysis for postoperative complications

Variable	*p*-value	Categories	Odds ratio	LCL	UCL	*p*-value (pair-wise)
ASA	< 0.001	III/IV vs II	1.419	1.211	1.663	< 0.001
		III/IV vs I	1.639	1.223	2.196	< 0.001
		II vs I	1.155	0.884	1.508	0.291
Age [10-year OR]	< 0.001		1.122	1.051	1.198	
Risk factors	< 0.001	Yes vs no	1.288	1.110	1.496	
Preoperative pain	0.003	Yes vs unknown	1.696	1.255	2.292	< 0.001
		Unknown vs no	0.613	0.448	0.841	0.002
		Yes vs no	1.040	0.891	1.215	0.615
Sex	0.010	Female vs male	1.329	1.069	1.652	
Defect size	0.013	III (> 3 cm) vs II (1.5–3 cm)	1.247	1.071	1.451	0.004
		III (> 3 cm) vs I (< 1.5 cm)	1.274	0.972	1.671	0.080
		II (1.5–3 cm) vs I (< 1.5 cm)	1.022	0.789	1.322	0.870
Mesh weight	0.013	≤ 50 g/m^2^ vs > 50 g/m^2^	1.213	1.041	1.413	
BMI [5-point OR]	0.027		1.111	1.012	1.220	
EHS classification	0.091	Medial vs lateral	0.833	0.703	0.986	0.033
		Lateral vs combined	1.117	0.935	1.334	0.224
		Medial vs combined	0.930	0.763	1.133	0.471
Fixation	0.375	No mesh fixation vs glue	0.454	0.186	1.109	0.083
		No mesh fixation vs suture	0.536	0.252	1.138	0.105
		No mesh fixation vs tacks	0.516	0.103	2.578	0.420
		Suture vs glue	0.847	0.519	1.383	0.507
		Tacks vs glue	0.881	0.196	3.960	0.868
		Suture vs tacks	0.962	0.231	4.002	0.958
Pore size	0.552	Small vs large	1.051	0.891	1.240	

*LCL* lower confidence limit, *UCL* upper confidence limit

No significant relation could be demonstrated between pore size and either intraoperative, general or postoperative complications.

The risk of complication-related reoperation was significantly associated with ASA classification (*p* = 0.002), age (*p* = 0.013), the presence of risk factors (*p* = 0.036) and tended to be associated with EHS classification (*p* = 0.062), preoperative pain (*p* = 0.066), pore size (*p* = 0.086) and mesh weight (*p* = 0.087). Accordingly, a higher ASA, a higher age, the presence of at least one risk factor, preoperative pain and low weight mesh were associated with a higher risk of reoperation. On the other hand, medial EHS classifications and smaller pore size (OR = 0.778 [0.585; 1.036]) were associated with a lower complication-related reoperation rate (Table 7).

**Table 7:** Multivariable analysis for complication-related reoperations

Variable	*p*-value	Categories	Odds ratio	LCL	UCL	*p*-value (pair-wise)
ASA	0.002	III/IV vs II	1.597	1.229	2.075	< 0.001
		III/IV vs I	1.659	1.017	2.707	0.043
		II vs I	1.039	0.660	1.635	0.868
Age [10-year OR]	0.013		1.151	1.030	1.286	
Risk factors	0.036	Yes vs no	1.303	1.017	1.669	
EHS classification	0.062	Medial vs combined	0.686	0.495	0.950	0.023
		Medial vs lateral	0.756	0.564	1.014	0.062
		Lateral vs combined	0.907	0.686	1.198	0.492
Preoperative pain	0.066	Yes vs no	1.277	0.976	1.671	0.075
		Yes vs unknown	1.532	0.952	2.466	0.079
		Unknown vs no	0.833	0.501	1.387	0.483
Pore size	0.086	Small vs large	0.778	0.585	1.036	
Mesh weight	0.087	≤ 50 g/m^2^ vs > 50 g/m^2^	1.243	0.969	1.595	
Sex	0.274	Female vs male	1.231	0.849	1.785	
BMI [5-point OR]	0.279		1.090	0.933	1.274	
Fixation	0.311	Tacks vs glue	6.833	0.927	50.347	0.059
		Suture vs tacks	0.353	0.084	1.478	0.154
		Suture vs glue	2.414	0.593	9.826	0.219
		No mesh fixation vs tacks	< 0.001	< 0.001	> 999.999	0.962
		No mesh fixation vs suture	< 0.001	< 0.001	> 999.999	0.965
		No mesh fixation vs glue	< 0.001	< 0.001	> 999.999	0.967
Defect size	0.510	III (> 3 cm) vs II (1.5–3 cm)	1.140	0.888	1.464	0.303
		III (> 3 cm) vs I (< 1.5 cm)	1.226	0.769	1.954	0.392
		II (1.5–3 cm) vs I (< 1.5 cm)	1.075	0.688	1.679	0.751

*LCL* lower confidence limit, *UCL* upper confidence limit

No association between pore size and recurrence was found at 1-year follow-up.

However, it was shown that medial EHS classifications (*p* < 0.001), female gender (*p* < 0.001), higher BMI (*p* = 0.004) and higher ASA (*p* = 0.038) were associated with a higher risk of recurrence. On the other hand, older age (*p* = 0.041) was associated with a lower risk of recurrence (Table 8).

**Table 8:** Multivariable analysis for recurrence at 1-year follow-up

Variable	*p*-value	Categories	Odds ratio	LCL	UCL	p-value (pair-wise)
EHS classification	< 0.001	Medial vs lateral	2.297	1.669	3.163	< 0.001
		Medial vs combined	1.554	1.079	2.239	0.018
		Lateral vs combined	0.676	0.458	1.000	0.050
Sex	< 0.001	Female vs male	1.915	1.308	2.803	
BMI [5-point OR]	0.004		1.279	1.083	1.509	
ASA	0.038	III/IV vs I	1.945	1.127	3.356	0.017
		III/IV vs II	1.396	1.006	1.937	0.046
		II vs I	1.394	0.867	2.239	0.170
Age [10-year OR]	0.041		0.888	0.792	0.995	
Preoperative pain	0.147	Yes vs no	0.741	0.549	1.000	0.050
		Unknown vs no	0.836	0.506	1.380	0.483
		Yes vs unknown	0.887	0.547	1.437	0.625
Defect size	0.203	III (> 3 cm) vs I (< 1.5 cm)	0.677	0.414	1.108	0.120
		III (> 3 cm) vs II (1.5–3 cm)	0.783	0.569	1.078	0.133
		II (1.5–3 cm) vs I (< 1.5 cm)	0.864	0.554	1.349	0.521
Mesh weight	0.217	≤ 50 g/m^2^ vs > 50 g/m^2^	0.820	0.598	1.124	
Fixation	0.467	No mesh fixation vs tacks	0.136	0.008	2.230	0.162
		No mesh fixation vs glue	0.224	0.026	1.939	0.174
		No mesh fixation vs suture	0.312	0.044	2.240	0.247
		Suture vs tacks	0.434	0.059	3.205	0.413
		Suture vs glue	0.716	0.288	1.780	0.472
		Tacks vs glue	1.648	0.185	14.685	0.654
Risk factors	0.577	Yes vs no	0.917	0.678	1.242	
Pore size	0.580	Small vs large	1.099	0.786	1.537	

*LCL* lower confidence limit, *UCL* upper confidence limit

The 1-year follow-up showed that older age (*p* < 0.001), larger defects (*p* < 0.001) and small-pore meshes (OR = 0.851 [0.737; 0.985]; *p* = 0.029) were associated with a lower risk of pain at rest (OR = 0.851 [0.737; 0.983]). In contrast, preoperative pain, postoperative complications, medial EHS classifications, greater BMI, female gender (each *p* < 0.001), and higher ASA classification (*p* = 0.001) were associated with an increased risk of pain at rest (Table 9).

**Table 9:** Multivariable analysis for pain at rest at 1-year follow-up

Variable	*p*-value	Categories	Odds ratio	LCL	UCL	*p*-value (pair-wise)
Age [10-year OR]	< 0.001		0.791	0.756	0.828	
Preoperative pain	< 0.001	Yes vs no	1.631	1.411	1.886	< 0.001
		Unknown vs no	1.616	1.296	2.015	< 0.001
		Yes vs unknown	1.010	0.831	1.227	0.923
Sex	< 0.001	Female vs male	1.688	1.424	2.000	
Defect size	< 0.001	III (> 3 cm) vs II (1.5–3 cm)	0.676	0.587	0.778	< 0.001
		III (> 3 cm) vs I (< 1.5 cm)	0.590	0.481	0.725	< 0.001
		II (1.5–3 cm) vs I (< 1.5 cm)	0.874	0.731	1.044	0.137
Postoperative complications	< 0.001	Yes vs no	1.651	1.274	2.140	
EHS classification	< 0.001	Medial vs lateral	1.293	1.132	1.478	< 0.001
		Medial vs combined	1.271	1.077	1.499	0.005
		Lateral vs combined	0.982	0.837	1.152	0.826
BMI [5-point OR]	< 0.001		1.144	1.062	1.233	
ASA	0.001	II vs I	1.416	1.174	1.708	< 0.001
		III/IV vs I	1.417	1.130	1.778	0.003
		III/IV vs II	1.001	0.866	1.157	0.990
Pore size	0.029	Small vs large	0.851	0.737	0.983	
Mesh weight	0.271	≤ 50 g/m^2^ vs > 50 g/m^2^	0.929	0.815	1.059	
Risk factors	0.379	Yes vs no	1.059	0.932	1.203	
Fixation	0.894	No mesh fixation vs glue	0.773	0.397	1.503	0.448
		Suture vs glue	0.877	0.557	1.381	0.571
		No mesh fixation vs suture	0.881	0.537	1.445	0.616
		Tacks vs glue	0.777	0.174	3.472	0.742
		Suture vs tacks	1.128	0.270	4.708	0.869
		No mesh fixation vs tacks	0.994	0.220	4.498	0.994

*LCL* lower confidence limit, *UCL* upper confidence limit

In regard to pore size, this result (OR = 0.851) would correspond to pain at rest at follow-up in 51 out of 1000 operations with small-pore size meshes compared to 59 out of 1000 operations with large-pore size meshes (prevalence 5.51%).

A similar relation was confirmed in the 1-year follow-up analysis for pain on exertion.

Again, older age, larger defects and small-pored meshes (OR = 0.815 [0.731; 0.909]) were associated with a lower risk of pain on exertion (*p* < 0.001 each). Preoperative pain, higher BMI, postoperative complications, female gender and medial EHS classifications were associated with a higher risk of pain on exertion (*p* < 0.001 each) (Table 10).

**Table 10:** Multivariable analysis for pain at exertion at 1-year follow-up

Variable	*p*-value	Categories	Odds ratio	LCL	UCL	*p*-value (pair-wise)
Age [10-year OR]	< 0.001		0.723	0.699	0.748	
Sex	< 0.001	Female vs male	1.675	1.466	1.915	
Preoperative pain	< 0.001	Yes vs no	1.527	1.373	1.699	< 0.001
		Unknown vs no	1.421	1.200	1.682	< 0.001
		Yes vs unknown	1.075	0.923	1.252	0.353
Defect size	< 0.001	III (> 3 cm) vs II (1.5–3 cm)	0.699	0.628	0.778	< 0.001
		III (> 3 cm) vs I (< 1.5 cm)	0.596	0.511	0.696	< 0.001
		II (1.5–3 cm) vs I (< 1.5 cm)	0.853	0.745	0.976	0.021
BMI [5-point OR]	< 0.001		1.165	1.100	1.234	
Postoperative complications	< 0.001	Yes vs no	1.701	1.387	2.087	
EHS classification	< 0.001	Medial vs lateral	1.266	1.144	1.402	< 0.001
		Medial vs combined	1.293	1.139	1.468	< 0.001
		Lateral vs combined	1.021	0.904	1.154	0.737
Pore size	< 0.001	Small vs large	0.815	0.731	0.909	
ASA	0.088	II vs I	1.146	1.005	1.306	0.042
		III/IV vs II	0.940	0.839	1.054	0.292
		III/IV vs I	1.077	0.913	1.271	0.378
Fixation	0.470	No mesh fixation vs suture	0.735	0.491	1.099	0.133
		No mesh fixation vs glue	0.672	0.396	1.141	0.141
		No mesh fixation vs tacks	0.734	0.241	2.232	0.586
		Suture vs glue	0.914	0.644	1.299	0.618
		Tacks vs glue	0.915	0.306	2.735	0.874
		Suture vs tacks	0.999	0.353	2.824	0.999
Risk factors	0.693	Yes vs no	0.980	0.888	1.082	
Mesh weight	0.729	≤ 50 g/m^2^ vs > 50 g/m^2^	1.018	0.922	1.123	

*LCL* lower confidence limit, *UCL* upper confidence limit

In terms of pore size, this result (OR = 0.815) would correspond to pain on exertion at follow-up in 93 out of 1000 operations with small-pore meshes compared to 112 out of 1000 operations with large-pore meshes (prevalence 10.24%).

The analysis of the pain requiring treatment at the 1-year follow-up shows that the pore size of the mesh has no significant relation to this outcome variables. Age, gender, defect size, preoperative pain, EHS classification, ASA status, BMI and postoperative complications (each *p* < 0.001) were significantly associated with pain requiring treatment at 1-year follow-up. Again, older age and larger defects were associated with a lower risk of pain requiring treatment. On the other side, preoperative pain, medial EHS classification, higher ASA classification, higher BMI, female gender and postoperative complications were associated with an increased risk of pain requiring treatment at 1-year follow-up (Table 11).

**Table 11:** Multivariable analysis for pain requiring treatment at 1-year follow-up

Variable	*p*-value	Categories	Odds ratio	LCL	UCL	*p*-value (pair-wise)
Age [10-year OR]	< 0.001		0.762	0.718	0.809	
Sex	< 0.001	Female vs male	2.209	1.795	2.718	
Defect size	< 0.001	III (> 3 cm) vs I (< 1.5 cm)	0.478	0.365	0.626	< 0.001
		III (> 3 cm) vs II (1.5–3 cm)	0.605	0.498	0.736	< 0.001
		II (1.5–3 cm) vs I (< 1.5 cm)	0.789	0.629	0.991	0.042
Preoperative pain	< 0.001	Yes vs no	1.762	1.441	2.155	< 0.001
		Unknown vs no	1.660	1.225	2.250	0.001
		Yes vs unknown	1.062	0.815	1.383	0.657
EHS classification	< 0.001	Medial vs lateral	1.598	1.339	1.907	< 0.001
		Medial vs combined	1.406	1.128	1.752	0.002
		Lateral vs combined	0.880	0.707	1.095	0.252
ASA	< 0.001	III/IV vs I	2.217	1.623	3.029	< 0.001
		II vs I	1.728	1.327	2.251	< 0.001
		III/IV vs II	1.283	1.061	1.552	0.010
BMI [5-point OR]	< 0.001		1.220	1.111	1.340	
Postoperative complications	< 0.001	Yes vs no	1.757	1.257	2.455	
Pore size	0.120	Small vs large	0.857	0.706	1.041	
Fixation	0.142	Suture vs glue	0.584	0.352	0.971	0.038
		No mesh fixation vs suture	1.370	0.793	2.369	0.259
		No mesh fixation vs glue	0.801	0.383	1.673	0.555
		Tacks vs glue*	–	–	–	0.952
		No mesh fixation vs tacks*	–	–	–	0.953
		Suture vs tacks*	–	–	–	0.955
Mesh weight	0.215	≤ 50 g/m^2^ vs > 50 g/m^2^	0.894	0.749	1.067	
Risk factors	0.347	Yes vs no	0.921	0.775	1.094	

*LCL* lower confidence limit, *UCL* upper confidence limit

* No valid information available as categories are not sufficiently populated

### Standardized differences of patients with and without follow-up information

**Fig. 2:**
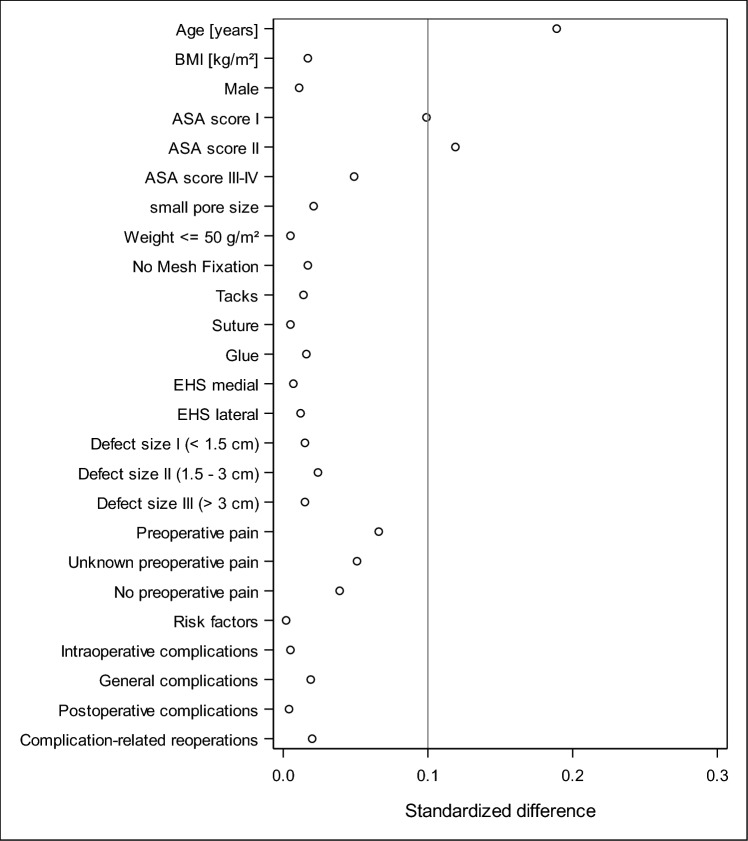
Scatter plot—standardized differences between patient with and without follow-up

Figure 2 shows the results of the standardized differences for patients with (*n* = 22,141) and without (*n* = 12,845) follow-up information. Standardized differences above a value of 10% were found only for age and ASA categories. The patients in the analysis population were on average 2.8 years older and had more frequently ASA II classification.

## Discussion

In order to reduce mesh-related side effects such as foreign body sensation, chronic pain or mesh shrinkage following hernioplasty, research has focused on improving the biocompatibility of meshes. Experimental data show that large-pored meshes reduce foreign body reaction, inflammation and scar bridging and thus improve mesh integration [16, 17, 21, 22]. However, clinical data on the effect of mesh porosity on the outcome of hernioplasty are still limited [2, 3, 14, 25]. This Herniamed registry study evaluates the largest patient cohort (22,141 patients) to date with regard to the relation of mesh porosity in polypropylene meshes to the outcome of Lichtenstein inguinal hernioplasty. However, patient and surgical characteristics differed significantly between the small- and large-pore mesh groups, which is related to the design of the registry study reflecting everyday care rather than selective bias. In general, a higher proportion of patients (69%) were treated with large-pored polypropylene meshes in this study. Furthermore, the patients who had large-pore polypropylene mesh repair were older, had more comorbidities and risk factors, and had larger hernia defects. In addition, a higher proportion of heavyweight meshes (69.3%) was used in large-pore mesh repair. The reason for the apparent preference for large-pored meshes in the treatment of elderly patients and rather difficult hernia situations in everyday care remains speculation. It might reflect the positive characteristics attributed to large-pored meshes from experimental studies.

Chronic postoperative pain as one of the primary outcome measures of inguinal hernia surgery was a main focus of attempts to improve the biocompatibility of meshes.

In this context, large-pored meshes are intended to enable tissue ingrowth, provide better mesh integration, maintain the elasticity of the implant and reduce scar formation [23, 24].

In our study, patients in the large-pore mesh group had a higher risk of both pain at rest and pain on exertion at 1-year follow-up. In contrast, in a randomized clinical trial of 321 patients, O'Dwyer et al. reported less chronic pain but an increase in hernia recurrence in the group treated with large-pore lightweight mesh for inguinal hernia repair at 12-month follow-up [3]. Similarly, in a randomized study of 590 patients who underwent Lichtenstein repair, Bringman et al. described an improvement in pain and discomfort in the group treated with a large-pore lightweight mesh, without affecting the recurrence rate at 3-year follow-up [25]. In both studies, large-pore lightweight meshes were used, while in our study, the majority of patients in the large-pore group received heavyweight meshes. Despite the reported benefits in terms of postoperative pain in the early postoperative course for lightweight meshes, these results are controversial in the literature with regard to the long-term effect [9–12, 15, 26–30].

Krauß et al. reported in a questionnaire study of the Danish Hernia Registry with 1782 patients that large-pore light weight mesh repair does not reduce chronic pain, but increases the recurrence rate compared to small-pore, heavy mesh repair [14]. Consistent with the results of our study, Nikkolo et al. saw more patients with pain in the large-pore mesh group at 3-year follow-up in a randomized trial of 128 patients [2].

Regarding the clinical relevance of our results, it should be noted that even very small differences can be significant due to the relatively large number of cases.

The described differences in postoperative pain between the groups with large-pore and small-pore mesh repair were not found in the analysis of pain requiring treatment at 1-year follow-up in our study.

It is known that the development of chronic postoperative pain after inguinal hernia repair is multifactorial [7]. In our study, in addition to large-pored meshes, younger age, smaller defects, preoperative pain, postoperative complications, medial EHS classifications, higher BMI, and female gender were associated with a higher risk of pain at rest and on exertion. In this context, young age, female gender, preoperative pain and postoperative complications have been identified in previous studies as risk factors for chronic postoperative pain after inguinal hernia repair [31].

Considering the multifactorial genesis and the size of the study population, a cautious conclusion regarding clinical relevance could be that the use of large-pore polypropylene mesh did not improve chronic postoperative pain in our study.

The analysis of the recurrence rate as a primary outcome measure showed no association to the mesh pore size at 1-year follow-up in our study. In contrast, both O'Dwyer et al. and Krauß et al. reported increased recurrence rates after anterior inguinal hernia repair with large-pore lightweight meshes in the above-mentioned studies [3, 14]. Experimental studies have shown that lightweight meshes with a large-pore size may lack structural stability and, therefore, have a tendency for mesh shrinkage [16]. In line with these findings, some studies and meta-analyses reported increased recurrence rates in lightweight mesh inguinal hernia repair with both anterior and laparoendoscopic techniques [30, 32, 33]. However, these results are not consistent in the literature, as further meta-analyses did not confirm increased recurrence rates for open inguinal hernia repair with light weight mesh [10, 11, 34].

In our study, the factors associated with a higher risk of recurrence were medial EHS classifications, female gender, higher BMI and higher ASA score, which is consistent with risk factors reported in the literature [35, 36].

When evaluating intraoperative, general or postoperative complications after Lichtenstein repair, no differences were found between the large-pore and small-pore mesh groups in this study. However, a lower risk of complication-related reoperations was associated with small-pore mesh repair. In the literature to date, there is no data on the influence of mesh porosity on intraoperative or postoperative complications.

Register studies have several limitations, including the present study. The patients enrolled in the registry are heterogeneous, as is the daily practice of hernia surgery. The analysis of register data for certain outcome parameters can, therefore, hardly be performed on heterogeneous groups. In addition, participation in the Herniamed registry is voluntary. Not all hospitals and surgeons in the participating countries include patients in the registry, which could imply a certain bias. Furthermore, a relevant proportion of patients with missing follow-up data could not be included in the analysis of this study. However, standardized differences of more than 10% between the study population and the excluded patients with incomplete follow-up data were only found for age and ASA categories. Therefore, there should only be a limited selection bias in terms of completeness of follow-up. Nevertheless, the follow-up results should be interpreted with caution.

In summary, this Herniamed registry study, investigating the association of mesh porosity in polypropylene meshes to the outcome of Lichtenstein inguinal hernioplasty, found no significant relation between mesh pore size and recurrence rate, intraoperative, general or postoperative complications as well as pain requiring treatment at 1-year follow-up.

Taking into account the potential influence of other patient and surgical characteristics, a significant effect of mesh pore size on complication-related reoperations (tendency), on pain at rest and on pain on exertion was demonstrated at 1-year follow-up.

In general, it should be noted that due to the relatively large number of cases, even very small differences can be significant, but their clinical significance in this context should be interpreted with caution.

In a cautious conclusion regarding clinical relevance, the present study could not demonstrate an advantage of large-pore meshes for the outcome of Lichtenstein inguinal hernioplasty.

## Data Availability

The data that support the findings of this study are available from the corresponding author upon reasonable request.
